# Real-world Data on Treatment Patterns and Bleeding in Cancer-associated Thrombosis: Data from the TROLL Registry

**DOI:** 10.1055/s-0044-1782219

**Published:** 2024-03-26

**Authors:** Zygimantas Zaboras, Camilla Tøvik Jørgensen, Andreas Stensvold, Heidi Hassel Pettersen, Aleksandra Galovic Grdinic, Sigrid Kufaas Brækkan, Waleed Ghanima, Mazdak Tavoly

**Affiliations:** 1 Institute of Clinical Medicine, University of Oslo, Oslo, Norway; 2 Department of Emergency Medicine, Østfold Hospital, Sarpsborg, Norway; 3 Department of Oncology, Østfold Hospital, Sarpsborg, Norway; 4 Department of Research, Østfold Hospital, Sarpsborg, Norway; 5 Clinic of Internal Medicine, Østfold Hospital, Sarpsborg, Norway; 6 Division of Internal Medicine, University Hospital of North Norway, Tromsø, Norway; 7 Department of Clinical Medicine, Thrombosis Research Group (TREC), UiT – The Arctic University of Norway, Tromsø, Norway; 8 Department of Hematology, Oslo University Hospital and Institute of Clinical Medicine, University of Oslo, Oslo, Norway; 9 Department of Medicine, Sahlgrenska University Hospital, Gothenburg, Sweden

**Keywords:** cancer-associated thrombosis, venous thromboembolism, bleeding, anticoagulants, direct oral anticoagulants, low molecular weight heparin

## Abstract

**Background** International guidelines are increasingly recommending direct oral anticoagulants (DOACs) as the first-line treatment for cancer-associated thrombosis (CAT). However, data regarding treatment patterns and adherence to guidelines in patients with CAT are scarce.

**Objectives** This study aimed to explore anticoagulant treatment patterns in patients with CAT and to calculate the incidence rates of bleeding events.

**Methods** Patients ≥18 years with active cancer and a first-time venous thromboembolism between 2005 and 2020 were identified through the Venous **T** hrombosis **R** egistry in Østf **OL** d Hospita **L** . Outcome measures were patterns of anticoagulant treatment during the study period and bleeding events. We calculated overall incidence rates per 100 person-years and 6- and 12-month cumulative incidence of major and clinically relevant nonmajor bleeding (CRNMB) during anticoagulant treatment.

**Results** Median age of 842 CAT patients at the time of thrombosis was 69 years (interquartile range 61–77), and 443 (52.6%) were men. In total, 526 patients (62.5%) had pulmonary embolism and 255 (30.3%) had deep vein thrombosis. Low molecular weight heparin (LMWH) was prescribed to 713 (85.8%) patients, whereas 64 (7.7%) received DOACs and 54 (6.5%) received vitamin K antagonists as the initial anticoagulant treatment. Prescription of DOACs as initial treatment increased from 3.0% in 2013/2014 to 18.0% in 2019/2020. The incidence rate of major bleeding was 6.9 (95% confidence interval [CI] 5.2–9.2) and 10.1 (95% CI 8.0–12.9) in CRNMB.

**Conclusion** Most patients were treated with LMWH. However, a gradual shift in treatment toward DOACs was observed. Overall, bleeding complications were rare and comparable to those reported in randomized trials.

## Background

Venous thromboembolism (VTE) is a frequently occurring complication in patients with cancer. Over the past two decades, the incidence of VTE in patients with active cancer has increased three-fold, whereas for patients treated with chemotherapy or targeted cancer treatment, it has increased up to six-fold. 1 Patients with cancer-associated thrombosis (CAT) are at a higher risk of mortality and morbidity and tend to consume more health care resources than cancer patients without VTE. 2 3 4 5 Moreover, patients with CAT have an increased risk of recurrent VTE, yet at the same time they have an increased risk of bleeding compared to VTE patients without cancer. 6 In this context, the care of CAT patients is a particularly vexing clinical problem. Traditionally, CAT has been treated with low molecular weight heparin (LMWH). 7 More recently, several studies have confirmed the efficacy and safety of direct oral anticoagulants (DOACs) in patients with CAT. 8 9 10 11 Consequently, several DOACs have been approved and recommended by international guidelines as an alternative or first-line treatment option for patients with CAT. 12 13 14 15 16 However, several uncertainties remain regarding the use of DOACs in CAT, including possible drug–drug interactions and the higher risk of bleeding in gastrointestinal and genitourinary malignancies. Therefore, physicians may be reluctant to prescribe DOACs to patients with CAT. Furthermore, limited data exist regarding physicians' adherence to clinical practice guidelines for the treatment of patients with CAT. Accordingly, the primary aim of this study was to assess anticoagulant treatment patterns in patients with CAT during the period 2005 to 2020. The secondary aim was to assess the incidence of bleeding events.

## Materials and Methods

### Study Design and Population

This was a cohort study based on data from the Venous **T** hrombosis **R** egistry in Østf **OL** d Hospita **L** (TROLL registry). TROLL is a single-center VTE registry in Østfold county, Norway. The registry, which was established in 2005, includes prospectively consecutive patients diagnosed, treated, or followed up for VTE at Østfold Hospital. Detailed information about the TROLL registry has been described previously. 17

Patients ≥18 years with a diagnosis of CAT registered in TROLL between January 2005 and May 2020 were eligible for study inclusion. Patients were required to have a first-time VTE diagnosis, symptomatic or incidental, that was radiologically verified by computed tomography pulmonary angiography, ventilation/perfusion (V/Q) scintigraphy, compression ultrasound, abdominal CT, magnetic resonance imaging, or autopsy. CAT was defined as a venous thrombotic event including deep vein thrombosis (DVT), pulmonary embolism (PE) (with or without DVT), splanchnic vein thrombosis, or upper extremity deep vein thrombosis (UEDVT), in patients with active cancer. Active cancer was defined as a cancer diagnosis confirmed within the previous 6 months or ongoing anticancer treatment, cancer recurrence with local or distant spreading, or hematological malignancy that was not in complete remission. 15 Exclusion criteria were previous VTE diagnosis, cerebral venous sinus thrombosis or superficial thrombosis, and patients without active cancer or diagnosed with cancer after the VTE diagnosis. The registry is continuously reviewed and updated for the present study for completion of missing data and outcomes concerning patients with CAT.

### Study Variables

Demographic, clinical, and cancer-related data collected included age, sex, body mass index calculated in kg/m ^2^ , smoking status, risk factors for VTE, localization of VTE, and type and site of malignancy. Cancer diagnoses were registered according to the International Classification of Diseases, 10th Revision, Clinical Modification and grouped into the following 15 categories: ear, nose, and throat; upper gastrointestinal tract (esophagus, stomach, and small intestine); lower gastrointestinal tract (colon, rectum, and anus); hepatobiliary and pancreatic; respiratory or mediastinal; skin, bone, and other connective tissues; breast; male genital organs (prostate, testicles, and penis); gynecological (cervix, uterus, ovaries, vagina, and vulva); urinary tract (kidneys, bladder, and urethra); central nervous system (eye, brain, and spinal cord); endocrine organs (thyroid, adrenal, and others); hematological (lymphoid, hematopoietic, or related tissue); separate categories for secondary or unspecified primary cancer and multiple primary cancers from different sites.

### Study Outcomes

The primary aim of this study was to assess the treatment patterns of anticoagulant agents during the period 2005 to 2020. Anticoagulant agents were categorized as follows: LMWH (dalteparin and enoxaparin), vitamin K antagonist (VKA; warfarin), and DOAC (rivaroxaban, edoxaban, apixaban, and dabigatran). Anticoagulant treatment was categorized as initial or secondary. Initial treatment was defined as the first anticoagulant treatment after CAT diagnosis lasting for more than 2 weeks. Secondary treatment was defined as the anticoagulant agent to which the patients switched from the initial treatment if switching was performed. We did not consider treatments administered for less than 2 weeks as the initial treatment because many patients received LMWH as a bridging therapy to the initial treatment phase or for other reasons. There was no restriction on the total treatment duration.

The secondary aim was to assess the incidence of major and clinically relevant nonmajor bleeding (CRNMB) events during anticoagulant therapy. Bleeding events were identified at the follow-up visits at the thrombosis outpatient clinic or by reviewing patients' electronic medical records. Bleeding events were classified according to the Control of Anticoagulation Subcommittee of the International Society on Thrombosis and Haemostasis classification as major bleeding or CRNMB. 18 19 Follow-up time started from the date of CAT diagnosis to the date of a first bleeding event, the last day of anticoagulant treatment, date of death, or the end of the study period (May 7, 2020), whichever occurred first.

### Statistical Analysis

Baseline characteristics are presented according to the localization of the thrombosis (PE, DVT, splanchnic, and UEDVT). Categorical variables are expressed as frequencies and percentages, whereas continuous variables are expressed as medians with corresponding interquartile ranges (IQRs). Anticoagulation treatment patterns for initial and secondary treatment were analyzed throughout the study period: LMWH and VKA from 2005 to 2020 and DOACs from 2013 to 2020. Prescription of DOAC, as initial treatment, was assessed separately by a subgroup analysis of the most common cancer groups, and in the cancers that, according to the literature, are associated with a higher risk of bleeding (upper and lower gastrointestinal tract, and genitourinary malignancies). 16 Incidence rates of bleeding were computed by dividing the number of events by the person-time at risk and expressed per 100 person-years with 95% confidence intervals (CIs). Incidence rates were analyzed separately for major bleeding and CRNMB and stratified according to most common cancer groups and those according to literature associated with higher risk of bleeding (as described above). CRNMB events were not censored in the analysis of major bleeding if CRNMB occurred before major bleeding. When analyzing CRNMB, major bleeding was censored if it occurred prior to the CRNMB event and the anticoagulant treatment was changed due to the major bleeding event. Cumulative incidences, with 95% CIs of bleeding events at 6 and 12 months were calculated using 1-Kaplan–Meier analyses. As competing risk by death may affect cumulative incidence rates for major bleeding and CRNMB, all conducted analyses considered death as a competing risk using the Fine–Gray regression model. A Poisson regression model with incidence rate ratios was used for time-trend analyses, and incidence rates were calculated for 2-year periods between 2005 and 2020 as the number of overall incident bleeding events divided by the time at risk in each period. The choice of 2-year periods was convenient and reduced the year-by-year fluctuations. All statistical analyses were conducted using Stata for Windows (StataCorp. 2021; Stata Statistical Software: Release 17. StataCorp LLC, College Station, TX).

### Ethics and Approval

The Regional Ethics Committee (reference number 267223) approved the study for participants who provided written informed consent and waived consent for deceased subjects.

## Results

In total, 4,673 patients with a first-time VTE were registered in TROLL between January 2005 and May 2020. Of these, 842 patients (18%) were diagnosed with CAT and were included in this study. Median age was 69 years (IQR 61–77 years), and 443 (52.6%) were men. Patient characteristics and risk factors associated with VTE are displayed in Table 1 . The most common VTE diagnosis was PE in 526 patients (62.5%), followed by DVT in 255 (30.3%) patients ( Table 1 ).

**Table 1:** Patient characteristics and risk factors

	Total VTE ( *n* = 842)	PE ( *n* = 526)	DVT ( *n* = 255)	Splanchnic ( *n* = 37)	UEDVT ( *n* = 24)
**Characteristics**					
Age, years, median (IQR)	69 (61–77)	69 (61–76)	69 (61–78)	64 (59–71)	60.5 (54–70)
Men, *n* (%)	443 (52.6)	273 (51.9)	140 (54.9)	17 (46.0)	13 (54.2)
Symptomatic VTE, *n* (%)	598 (71.0)	339 (64.4)	242 (94.9)	NA a	17 (70.8)
Family history of VTE, *n* (%)	24 (2.9)	13 (2.5)	8 (3.1)	2 (5.4)	1 (4.2)
Known thrombophilia, *n* (%)	5 (0.6)	4 (0.8)	1 (0.4)	0 (0)	0 (0)
Smoking, *n* (%)	136 (17.4) b	81 (16.2)	39 (17.0)	11 (35.5)	5 (23.8)
**Risk factors**					
Surgery, *n* (%)	177 (21.0)	111 (21.1)	52 (20.4)	8 (21.6)	6 (25.0)
Immobilization, *n* (%)	48 (5.7)	32 (6.1)	14 (5.5)	0 (0)	2 (8.3)
Trauma, *n* (%)	13 (1.5)	5 (1.0)	6 (2.4)	0 (0)	2 (8.3)
Contraception and hormone replacement therapy, *n* (%)	9 (2.3)	5 (2.0)	3 (2.6)	1 (5.0)	0 (0)
Long-haul (>4 h) flights, *n* (%)	14 (1.7)	9 (1.7)	4 (1.6)	0 (0)	1 (4.2)
BMI over 30, *n* (%)	294 (42.4) c	171 (38.4)	100 (52.4)	14 (40.0)	9 (40.9)

Abbreviations: BMI, body mass index (calculated in kg/m ^2^ ); DVT, deep vein thrombosis; IQR, interquartile range; NA, not applicable; PE, pulmonary embolism; UEDVT, upper extremity deep vein thrombosis; VTE, venous thromboembolism.

a Symptoms in patients with splanchnic VTE were unavailable.

b Missing data in 62 patients.

c Missing data in 149 patients.

### Cancer Groups

**Fig. 1:**
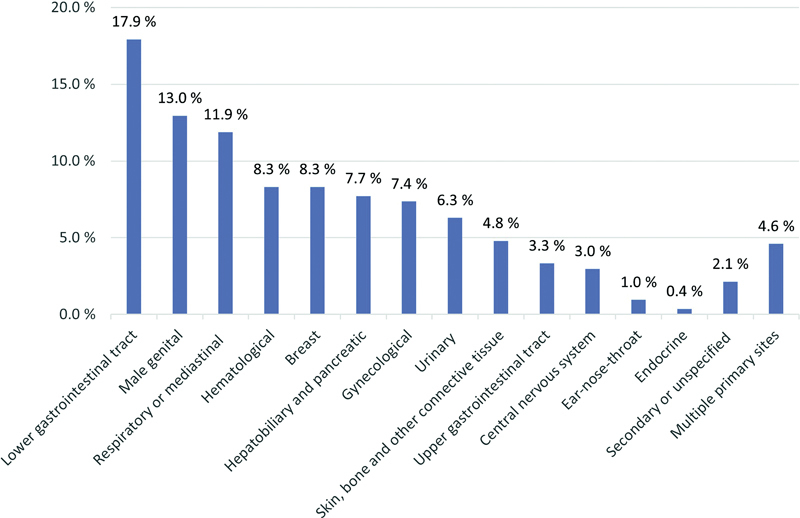
Distribution of cancer sites.

The five most common cancers were lower gastrointestinal ( *n* = 151, 17.9%), male genital ( *n* = 109, 13.0%), respiratory or mediastinal ( *n* = 100, 11.9%), hematological ( *n* = 70, 8.3%), and breast ( *n* = 70, 8.3%; Fig. 1 ). Overall, 357 patients (42.4%) had metastatic disease. Further characteristics according to cancer site are summarized in Supplementary Table S1 .

### Treatment Patterns

**Fig. 2:**
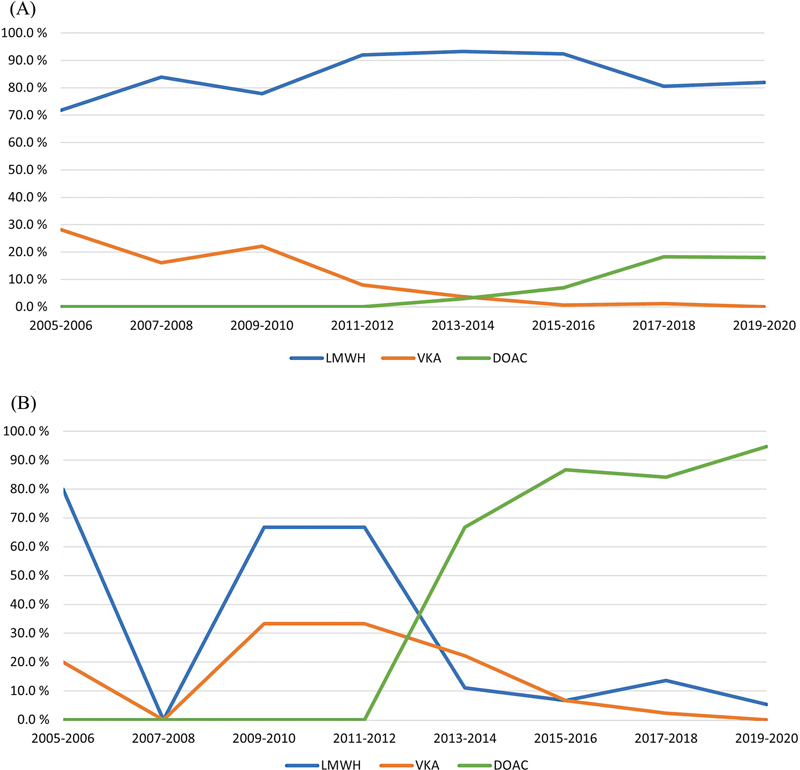
Time-trend analysis of initial ( **A** ) and secondary anticoagulant treatment ( **B** ) during 2005 to 2020. None of the patients was prescribed secondary anticoagulant treatment in 2007 to 2008. DOAC, direct oral anticoagulant; LMWH, low molecular weight heparin; VKA, vitamin K antagonist.

Initial anticoagulant treatment consisted of LMWH in 713 (85.8%), DOAC in 64 (7.7%), and VKA in 54 (6.5%) patients during the study period. Median duration of initial anticoagulant treatment was 144.5 days (IQR 61–221 d). Similar treatment patterns were observed when restricting the period from 2013 to 2020 (when DOACs became available); LMWH was prescribed in 482 (87.0%), DOACs in 64 (11.6%), and VKAs in 8 (1.4%) patients. As expected, treatment patterns shifted during the study period. VKA prescriptions declined from 28.2 to 0% from 2005/2006 to 2019/2020, whereas DOAC prescription increased from 3.0% in 2013/2014 to 18.0% in 2019/2020 ( Fig. 2A ).

Most patients with initial LMWH treatment (598/713, 83.9%) continued on LMWH without switching to anticoagulant agents. Secondary anticoagulant treatment was prescribed in 139 (16.7%) patients. Of these, 110 (79.1%) patients switched from LMWH or VKA to DOACs and 21 (15.1%) patients switched from DOAC or VKA to LMWH. Median duration of secondary anticoagulant treatment was 128.5 days (IQR 48–219 d). Prescription of DOAC as secondary treatment increased from 66.7% (6/9) to 94.7% (54/57) from 2013 to 2020 ( Fig. 2B ).

**Fig. 3:**
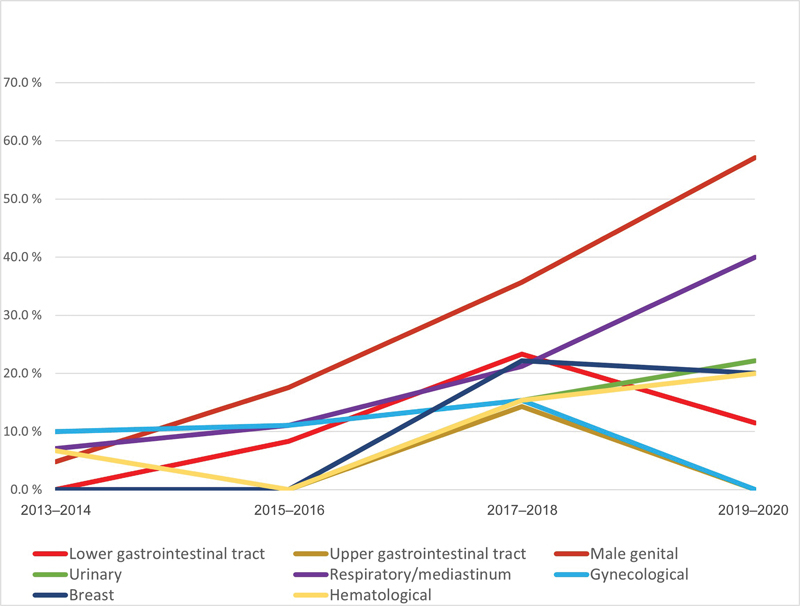
Proportion of patients receiving direct oral anticoagulants as initial treatment according to cancer site between 2013 and 2020.

The largest increase in DOAC prescription as initial treatment was observed in patients with cancer in male genital organs (from 4.8 to 57.1%) and in patients with respiratory or mediastinal cancers (from 7.1 to 40.0%). DOACs were prescribed to 23.3% with lower gastrointestinal tract, 15.4% with gynecological, and 14.3% with upper gastrointestinal tract cancers in 2017/2018. However, no patient with gynecological or upper gastrointestinal tract cancers was prescribed DOACs in 2019/2020, whereas only 11.5% of patients with lower gastrointestinal tract cancer received a DOAC ( Fig. 3 ).

### Bleeding

**Fig. 4:**
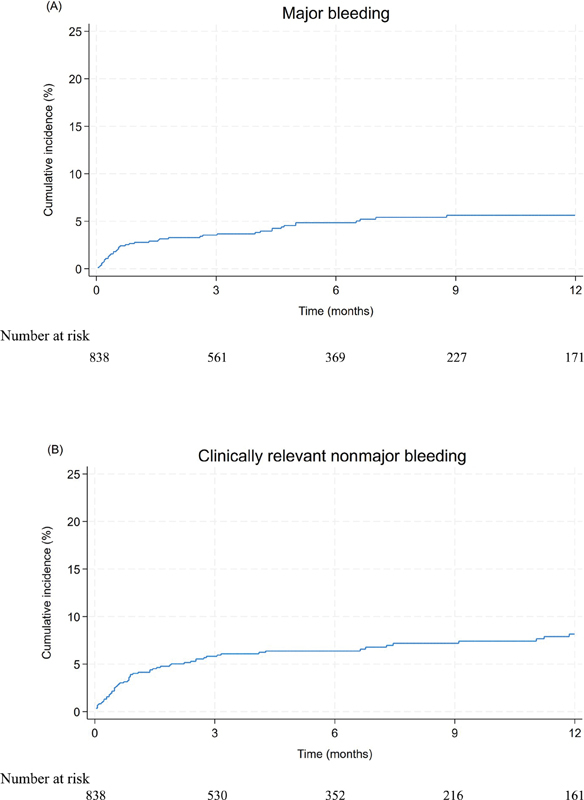
Cumulative incidence for major bleeding ( **A** ) and clinically relevant nonmajor bleeding ( **B** ) accounting for competing risk by death.

A total of 107 (12.7%) patients suffered from one or more bleeding events, of which 48 (44.9%) were major bleeding events and 59 (55.1%) were CRNMB events. The most common types of major bleeding were gastrointestinal ( *n* = 16, 33.3%), intracranial ( *n* = 7, 14.6%), abdominal ( *n* = 6, 12.5%), and trauma-related ( *n* = 4, 8.3%) ( Supplementary Table S4 and S5 , available in the online version). Analyzing bleeding events between 2013 and 2020 revealed that only three patients receiving DOACs experienced major bleeding. The overall incidence rate per 100 person-years was 6.9 (95% CI 5.2–9.2) for major bleeding and 10.1 (95% CI 8.0–12.9) for CRNMB ( Table 2 ). The overall 6- and 12-month cumulative incidences were 4.8% (95% CI 3.5–6.5%) and 5.6% (95% CI 4.1–7.5%) for major bleedings and 6.4% (95% CI 4.8–8.2%) and 8.2% (95% CI 6.3–10.4%) for CRNMB, respectively ( Table 2 and Fig. 4 ).

**Table 2:** Incidence rates per 100 person-years and cumulative incidences of bleeding in patients with cancer-associated thrombosis accounting for competing risk by death

	Incidence rates per 100 person years (95% CI)	6-month cumulative incidence, % (95% CI)	12-month cumulative incidence, % (95% CI)
Any bleeding	16.1 (13.3-19.5)	10.2 (8.2–12.4)	12.7 (10.3–15.3)
Major bleeding	6.9 (5.2–9.2)	4.8 (3.5–6.5)	5.6 (4.1–7.5)
CRNMB	10.1 (8.0–12.9)	6.4 (4.8–8.2)	8.2 (6.3–10.4)

Abbreviations: CI, confidence interval; CRNMB, clinically relevant nonmajor bleeding.

Any bleeding: major bleeding + CRNMB.

**Fig. 5:**
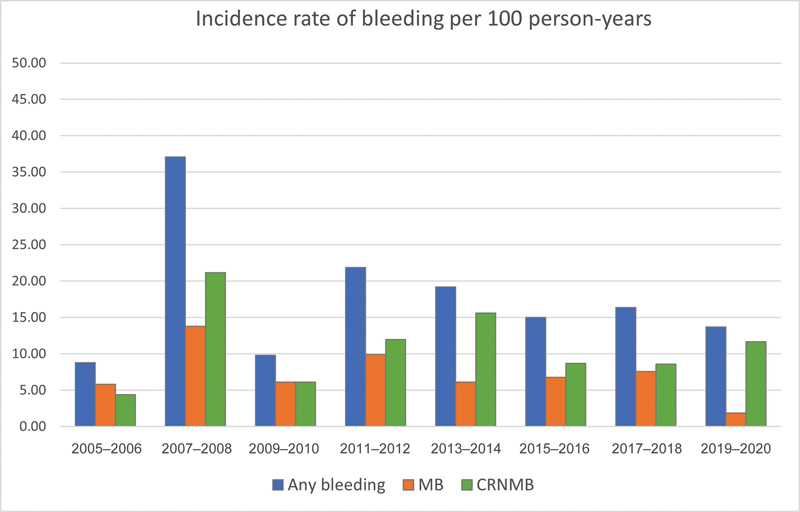
Time-trend analysis of bleeding incidence rates. Observed changes in incidence rates of bleeding (any bleeding, MB, or CRNMB) over the entire study period (2005–2020) were not statistically significant ( *p* -value was 0.69 for any bleeding, 0.49 for MB, and 0.50 for CRNMB). Any bleeding: Major bleeding + CRNMB. CRNMB, clinically relevant nonmajor bleeding; MB, major bleeding.

The time-trend analysis did not reveal a statistically significant change in the incidence rates of major bleeding and CRNMB during the study period ( Fig. 5 ). Major bleeding incidence rates decreased from 5.8 (95% CI 2.2–15.4) per 100 person-years in 2005 to 2006 to 1.9 (95% CI 0.3–13.3) per 100 person-years in 2019 to 2020. Incidence rates of CRNMB events increased in the beginning of the study period from 4.4 (95% CI 1.4–13.7) per 100 person-years in 2005 to 2006 to 21.2 (95% CI 8.0–56.5) in 2007 to 2008. However, a reduction of CRNMB events to 15.6 (95% CI 9.7–25.0) was observed in 2013 to 2014 and attenuated further to 11.7 (95% CI 5.3–26.1) per 100 person-years in 2019 to 2020 ( Fig. 5 ).

The rates of bleeding differed across cancer sites. Cumulative incidence of any bleeding at 6 months was 22.0% (95% CI 11.8–34.3%) in urinary tract cancers, 15.3% (95% CI 7.5–25.6%) in gynecological cancers, 9.7% (95% CI 5.6–15.2%) in lower gastrointestinal tract cancers, 8.2% (95% CI 3.8–14.7%) in respiratory or mediastinal cancers, and 5.2% (95% CI 1.9–10.9%) in male genital cancers. Cumulative incidence of major bleeding at 6 months was 6.9% (95% CI 2.2–15.3%) in gynecological cancers, 6.3% (95% CI 3.1–11.1%) in lower gastrointestinal tract cancers, 5.9% (95% CI 1.6–14.7%) in urinary tract cancers, 2.3% (95% CI 0.4–7.2%) in male genital organ cancers, and 2.2% (95% CI 0.4–6.8%) in respiratory or mediastinal organs. Further results displaying major bleeding and CRNMB events with incidence rates and cumulative incidences according to cancer site are summarized in Supplementary Tables S2 and S3 .

## Discussion

This study provides real-world data regarding treatment patterns and bleeding complications in patients diagnosed with CAT. Throughout the study period, most patients were prescribed LMWH as the initial anticoagulant treatment. However, DOACs were the most prescribed agents for secondary treatment and are gaining ground as the first-line initial treatment. There were no statistically significant changes in the incidence of major bleeding over time.

Current international guidelines recommend LMWH or DOACs for initial anticoagulant treatment in patients with CAT. Recently, some guidelines have favored DOACs over LMWH as initial treatment, albeit with caution, in patients with gastrointestinal and genitourinary malignancies because of increased risk of bleeding. 12 16 However, a recent study showed that DOACs may also be safe in patients with gastrointestinal cancers. 20 In the present study, both in the entire study period and during 2013 to 2020, patients were predominantly prescribed LMWH (87%) as initial treatment and only 11.6% received DOACs. Several other studies have reported similar findings. 5 21 22 However, some studies have reported prescription frequencies of DOACs of up to 50% as initial treatment in patients with CAT. 23 24 25 A lower proportion of patients with gastrointestinal or genitourinary malignancies and defining active cancer as cancer diagnosed up to 5 years preceding the thrombosis may be possible explanations for the higher DOAC prescription rate in these studies. 23 24 25

When studying treatment patterns, we observed an increase in DOAC prescription, both as an initial and secondary anticoagulant treatment. Male genital cancers, mainly prostate cancer, were among the malignancies in which DOAC prescription, as initial treatment, increased the most. In contrast, DOAC prescription remained low in patients with lower and upper gastrointestinal tract and gynecological malignancies, particularly toward the end of the study period. Possible explanations for this observation might be the overall low prescription rate of DOACs in the present study and the addition of recently published data suggesting increased risk of bleeding in the aforementioned malignancies when DOACs are used. 12 16 In general, these observations indicate that clinical practice adheres to the most recent treatment guidelines for CAT. 12 16 Trends in treatment patterns have not been extensively reported. One study reported similar findings regarding increased DOAC prescription. 5 However, no data were presented for treatment patterns according to cancer sites.

Before the introduction of DOACs, patients with CAT rarely changed anticoagulant treatment during their cancer disease. In this study, we found that most patients continued with the initial treatment. However, a dramatic increase in DOAC prescription as secondary treatment was found after 2013, and by the end of the study period, most patients received DOACs as secondary treatment. To the best of our knowledge, only one previous study has explored both initial and secondary anticoagulant treatment patterns. 26

In the present study, the 6- and 12-month cumulative incidences of major bleeding were 4.8 and 5.6%, respectively, which are similar to the rates reported by others. 23 25 Although, higher cumulative bleeding incidences have been reported previously, these studies have included more patients with genitourinary, gastrointestinal, and intracranial malignancies or prescribed mainly VKA anticoagulation. 6 27 28

Bleeding incidence rates were stable over time. The increased incidence rate of CRNMB observed throughout the study period was not significant, probably due to random variations. Bleeding events occurred more frequently in the lower gastrointestinal tract, urinary tract, male genital, and respiratory or mediastinal malignancies, which is in accordance with previous studies. 25 29 However, compared with two recent studies, we observed higher 6-month cumulative incidences of major bleeding, particularly in patients with lower gastrointestinal tract (6.3 vs. 4.6%) and higher 6-month cumulative incidence of any bleeding in patients with respiratory malignancies (8.2 vs. 4.2%). 23 30 Defining active cancer as confirmed within the previous 5 years and prescribing VKA anticoagulation with a low targeted therapeutic range in these studies may be possible explanations for the observed differences. 23 30

This study has several strengths. All patients were identified through the TROLL registry, which is an ongoing and continuously updated registry that includes all patients managed for VTE at our center. In contrast to other regions and countries in which patients with CAT may be followed up by oncologists, most patients diagnosed with VTE, including CAT, are referred to the thrombosis outpatient clinical at Østfold hospital and thus included in the registry. Presumably, most patients diagnosed with CAT in the Østfold region during the study period were identified and included in this study. In addition, identifying bleeding events at in-person follow-up visits enables registration of most clinically significant bleeding complications related to anticoagulant treatment.

Our study also has some limitations. This study is based on a single-center registry, which may influence the generalizability of the results. Furthermore, although most patients were referred to the thrombosis outpatient clinic and thus registered in TROLL, we cannot exclude that some patients have been followed up in other settings or sought medical attention at other health care institutions outside the Østfold region, thus hampering our bleeding analyses. However, VTE patients, including those with CAT, living in the catchment area would likely be followed up at Østfold hospital. Any events diagnosed in other hospitals would therefore probably be recorded in the TROLL registry at a later visit. Distinguishing between initial and secondary treatment was difficult to assess in some cases because there were overlapping treatment periods, which may have led to imprecise registration of initial and secondary treatment. In addition, we could not perform analysis regarding risk factors for bleeding in CAT patients because the data were not registered in the TROLL registry.

## Conclusion

Most patients diagnosed with CAT were still treated with LMWH. However, a gradual shift toward increased prescription of DOACs was observed. DOACs were most often used in patients with male genital and respiratory or mediastinal malignancies. Incidence rates of bleeding events were stable over time.
