# Supplementary material for: Real-world Data on Treatment Patterns and Bleeding in Cancer-associated Thrombosis: Data from the TROLL Registry
Source: TH Open. 2024 Mar 26;8(1):e132–40. doi: 10.1055/s-0044-1782219 (PMC10965301; doi:10.1055/s-0044-1782219)
Supplement: Supplementary file 1 — Supplementary Material [file 10-1055-s-0044-1782219-s23110045.pdf]

Supplementary Table S1 Distribution of cancers with and without metastases based on International Classification of Diseases codes among patients with pulmonary embolism, deep vein thrombosis, splanchnic thrombosis and upper extremity deep vein thrombosis

		Total VTE ($n = 842$)	PE ($n = 526$)	DVT ($n = 255$)	Splanchnic ($n = 37$)	UEDVT ($n = 24$)
**Cancer site**						
Lower gastrointestinal tract, *n* (%)	Total	151 (17.9)	98 (18.6)	37 (14.5)	13 (35.1)	3 (12.5)
	Metastatic	85 (56.3)[a]	57 (58.2)	22 (59.5)	6 (46.2)	0 (0%)
Male genitalia[b], *n* (%)	Total	109 (13.0)	60 (11.4)	47 (18.4)	0 (0)	2 (8.3)
	Metastatic	38 (34.9)	23 (38.3)	14 (29.8)	0 (0)	1 (50.0)
Respiratory or mediastinal, *n* (%)	Total	100 (11.9)	72 (13.7)	21 (8.2)	1 (2.7)	6 (25.0)
	Metastatic	34 (34.0)	24 (33.3)	8 (38.1)	0 (0)	2 (33.3)
Hematological, *n* (%)	Total	70 (8.3)	45 (8.6)	23 (9.0)	0 (0)	2 (8.3)
	Metastatic	0 (0)	0 (0)	0 (0)	0 (0)	0 (0)
Breast, *n* (%)	Total	70 (8.3)	41 (7.8)	24 (9.4)	1 (2.7)	4 (16.7)
	Metastatic	42 (60.0)	26 (63.4)	14 (58.3)	0 (0)	2 (50.0)
Hepatobiliary and pancreatic, *n* (%)	Total	65 (7.7)	37 (7.0)	20 (7.8)	7 (18.9)	1 (4.2)
	Metastatic	39 (60.0)	23 (62.2)	10 (50.0)	5 (71.4)	1 (100.0)
Gynecological, *n* (%)	Total	62 (7.4)	35 (6.7)	24 (9.4)	2 (5.4)	1 (4.2)
	Metastatic	26 (41.9)	18 (51.4)	8 (33.3)	0 (0)	0 (0)
Urinary[c], *n* (%)	Total	53 (6.3)	35 (6.7)	13 (5.1)	5 (13.5)	0 (0)
	Metastatic	21 (39.6)	16 (45.7)	5 (38.5)	0 (0)	0 (0)
Skin, bone, and other connective tissues, *n* (%)	Total	40 (4.8)	24 (4.6)	13 (5.1)	0 (0)	3 (12.5)
	Metastatic	17 (42.5)	15 (62.5)	2 (15.4)	0 (0)	0 (0)
Upper gastrointestinal tract, *n* (%)	Total	28 (3.3)	21 (4.0)	4 (1.6)	2 (5.4)	1 (4.2)
	Metastatic	16 (57.1)	14 (66.7)	0 (0)	1 (50.0)	1 (100.0)
Central nervous system[d], *n* (%)	Total	25 (3.0)	15 (2.9)	10 (3.9)	0 (0)	0 (0)
	Metastatic	2 (8.0)	1 (6.7)	1 (10.0)	0 (0)	0 (0)
Ear–nose–throat, *n* (%)	Total	8 (1.0)	6 (1.1)	2 (0.8)	0 (0)	0 (0)
	Metastatic	4 (50.0)	3 (50.0)	1 (50.0)	0 (0)	0 (0)
Endocrine, *n* (%)	Total	3 (0.4)	2 (0.4)	0 (0)	0 (0)	1 (4.2)
	Metastatic	2 (66.7)	1 (50.0)	0 (0)	0 (0)	1 (100.0)
Secondary or unspecified, *n* (%)	Total[e]	18 (2.1)	9 (1,7)	8 (3.1)	1 (2.7)	0 (0)
	Metastatic	16 (88.9)	9 (100.0)	6 (75.0)	1 (100.0)	0 (0)
Multiple primary sites, *n* (%)	Total	39 (4,6)	25 (4,8)	9 (3.5)	5 (13.5)	0 (0)
	Metastatic	15 (40.5)[a]	11 (44.0)	2 (22.2)	2 (40.0)	0(0)

Abbreviations: DVT, deep vein thrombosis; PE, pulmonary embolism; UEDVT, upper extremity deep vein thrombosis; VTE, venous thromboembolism.
[a]Percentage of total cases per cancer site.
[b]Cancer in penis, prostate, and testicles.
[c]Cancer in kidneys, bladder, and urethra.
[d]Cancer in eye, brain, and spinal cord.
[e]Includes unspecified types of cancer without metastases.

 © 2024. The Author(s).

Supplementary Table S2 Incidence rates for bleeding types per 100 person-years according to cancer site

		Total ($n=107$, 95% CI)	Major bleeding ($n=48$, 95% CI)	Clinically relevant nonmajor bleeding ($n=59$, 95% CI)
**Cancer site**				
Lower gastrointestinal tract (95% CI)	Total	17.7 (11.8–26.3)	9.1 (5.3–15.7)	9.6 (5.6–16.5)
	Metastatic	17.5 (10.4–29.6)	8.4 (4.0–17.6)	11.3 (5.9–21.8)
Male genitalia[a] (95% CI)	Total	8.5 (4.7–15.4)	2.3 (0.7–7.1)	7.8 (4.2–14.4)
	Metastatic	26.5 (11.9–59.0)	8.7 (2.2–34.8)	22.3 (9.3–53.5)
Respiratory or mediastinal (95% CI)	Total	18.2 (10.1–32.9)	4.9 (1.6–15.1)	13.3 (6.6–26.5)
	Metastatic	27.1 (10.2–72.1)	6.5 (0.9–45.8)	20.3 (6.5–62.9)
Hematological (95% CI)	Total	7.4 (2.8–19.6)	3.7 (0.9–14.6)	5.5 (1.8–17.1)
	Metastatic	0 (0)	0 (0)	0 (0)
Breast (95% CI)	Total	12.1 (6.5–22.5)	4.7 (1.8–12.4)	7.3 (3.3–16.2)
	Metastatic	11.2 (4.7–27.0)	6.7 (2.2–20.9)	4.5 (1.1–18.0)
Biliary and pancreatic (95% CI)	Total	43.8 (22.8–84.2)	18.5 (7.7–44.4)	19.5 (7.3–51.9)
	Metastatic	72.6 (32.6–161.5)	29.9 (11.2–79.7)	24.2 (6.0–96.7)
Gynecological (95% CI)	Total	24.8 (13.3–46.0)	9.4 (3.5–25.0)	17.3 (8.3–36.4)
	Metastatic	19.7 (7.4–52.5)	4.8 (0.7–33.8)	14.8 (4.8–45.8)
Urinary tract[b] (95% CI)	Total	37.1 (21.1–65,4)	11.3 (4.2–30.1)	27.9 (14.5–53.6)
	Metastatic	49.1 (20.4–117.9)	28.7 (9.2–88.9)	29.6 (9.6–91.8)
Skin, bone and other connective tissues (95% CI)	Total	32.9 (16.4–65.7)	22.5 (10.1–50.1)	8.2 (2.1–32.8)
	Metastatic	57.3 (23.9–137.8)	36.6 (13.7–97.5)	11.5 (1.6–81.4)
Upper gastrointestinal tract (95% CI)	Total	42.9 (16.1–114.3)	10.2 (1.4–72.7)	32.2 (10.4–99.8)
	Metastatic	48.2 (12.0–192.5)	0 (0)	48.2 (12.0–192.5)
Multiple primary sites (95% CI)	Total	7.3 (2.4–22.7)	4.6 (1.2–18.5)	4.9 (1.2–19.5)
	Metastatic	4.5 (0.6–31.7)	4.5 (0.6–31.7)	4.5 (0.6–31.7)
Secondary or unspecified (95% CI)	Total	21.3 (3.0–151.1)	21.3 (3.0–151.1)	0 (0)
	Metastatic	26.9 (3.8–190.9)	26.9 (3.8–190.9)	0 (0)

Abbreviation: CI, confidence interval.
[a]Cancer in penis, prostate, and testicles.
[b]Cancer in kidneys, bladder, and urethra.

 © 2024. The Author(s).

Supplementary Table S3 Cumulative incidence rates of bleeding at 6 and 12 months according to cancer site

		Any bleeding ($n=107$)		Major bleeding ($n=48$)		CRNMB ($n=59$)	
		6-month cumulative incidence, % (95% CI; %)	12-month cumulative incidence, % (95% CI; %)	6-month cumulative incidence, % (95% CI; %)	12-month cumulative incidence, % (95% CI; %)	6-month cumulative incidence, % (95% CI; %)	12-month cumulative incidence, % (95% CI; %)
**Cancer site**							
Lower gastrointestinal tract	Total	9.7 (5.6–15.2)	12.8 (7.7–19.3)	6.3 (3.1–11.1)	6.3 (3.1–11.1)	4.9 (2.2–9.3)	8.1 (4.1–13.9)
	Metastatic	9.8 (4.6–17.4)	12.8 (6.5–21.2)	5.1 (1.6–11.5)	5.1 (1.6–11.5)	7.3 (3.0–14.3)	10.3 (4.8–18.3)
Male genitalia[a]	Total	5.2 (1.9–10.9)	10.3 (4.7–18.6)	2.3 (0.4–7.2)	4.0 (1.0–10.4)	5.0 (1.8–10.5)	8.5 (3.6–16.1)
	Metastatic	8.6 (2.2–20.7)	16.5 (5.9–31.7)	3.1 (0.2–13.6)	7.2 (1.2–20.5)	8.5 (2.2–20.5)	12.5 (3.9–26.4)
Respiratory or mediastinal	Total	8.2 (3.8–14.7)	11.0 (5.6–18.5)	2.2 (0.4–6.8)	3.5 (0.9–9.2)	6.1 (2.5–11.9)	7.6 (3.3–14.3)
	Metastatic	8.8 (2.3–21.1)	8.8 (2.3–21.1)	2.9 (0.2–13.0)	2.9 (0.2–13.0)	5.9 (1.1–17.2)	5.9 (1.1–17.2)
Hematological	Total	4.4 (1.2–11.3)	4.4 (1.2–11.3)	3.0 (0.6–9.2)	3.0 (0.6–9.2)	2.9 (0.6–9.1)	2.9 (0.6–9.1)
	Metastatic	0 (0)	0 (0)	0 (0)	0 (0)	0 (0)	0 (0)
Breast	Total	13.8 (6.8–23.3)	13.8 (6.8–23.3)	4.6 (1.2–11.6)	4.6 (1.2–11.6)	9.4 (3.8–18.0)	9.4 (3.8–18.0)
	Metastatic	10.5 (3.3–22.4)	10.5 (3.3–22.4)	5.2 (0.9–15.4)	5.2 (0.9–15.4)	5.4 (1.0–15.8)	5.4 (1.0–15.8)
Hepatobiliary and pancreatic	Total	12.3 (5.8–21.5)	15.0 (7.2–25.4)	6.2 (2.0–13.8)	8.6 (3.1–17.6)	6.3 (2.0–14.1)	6.3 (2.0–14.1)
	Metastatic	12.8 (4.7–25.2)	16.5 (6.6–30.3)	7.7 (2.0–18.7)	11.2 (3.5–24.0)	5.3 (1.0–15.6)	5.3 (1.0–15.6)
Gynecological	Total	15.3 (7.5–25.6)	19.2 (9.3–31.8)	6.9 (2.2–15.3)	6.9 (2.2–15.3)	10.0 (4.1–19.1)	14.0 (5.6–26.2)
	Metastatic	12.1 (3.0–27.8)	20.1 (5.6–40.9)	4.0 (0.3–17.0)	4.0 (0.3–17.0)	8.1 (1.4–22.6)	16.1 (3.5–36.9)
Urinary tract[b]	Total	22.0 (11.8–34.3)	22.0 (11.8–34.3)	5.9 (1.6–14.7)	5.9 (1.6–14.7)	18.1 (8.9–29.8)	18.1 (8.9–29.8)
	Metastatic	20.3 (6.3–40.0)	20.3 (6.3–40.0)	10.1 (1.7–27.6)	10.1 (1.7–27.6)	15.6 (3.8–34.6)	15.6 (3.8–34.6)
Skin, bone, and other connective tissues	Total	19.6 (8.5–34.0)	23.7 (10.9–39.2)	14.5 (5.2–28.2)	18.6 (7.4–33.8)	5.3 (1.0–15.8)	5.3 (1.0–15.8)
	Metastatic	34.1 (12.3–57.6)	34.1 (12.3–57.6)	27.5 (8.5–50.9)	27.5 (8.5–50.9)	5.9 (0.4–23.5)	5.9 (0.4–23.5)
Upper gastrointestinal tract	Total	12.2 (3.1–28.1)	23.9 (5.9–48.5)	4.3 (0.3–17.9)	4.3 (0.3–17.9)	7.9 (1.4–22.3)	20.5 (3.7–46.7)
	Metastatic	13.9 (2.3–35.8)	13.9 (2.3–35.8)	0 (0)	0 (0)	13.9 (2.3–35.8)	13.9 (2.3–35.8)
Multiple primary sites	Total	7.9 (2.0–19.1)	7.9 (2.0–19.1)	5.3 (1.0–15.6)	5.3 (1.0–15.6)	5.2 (0.9–15.4)	5.2 (0.9–15.4)
	Metastatic	7.1 (0.5–27.5)	7.1 (0.5–27.5)	7.1 (0.5–27.5)	7.1 (0.5–27.5)	7.1 (0.5–27.5)	7.1 (0.5–27.5)
Secondary or unspecified	Total	5.6 (0.4–22.4)	5.6 (0.4–22.4)	5.6 (0.4–22.4)	5.6 (0.4–22.4)	0 (0)	0 (0)
	Metastatic	6.3 (0.4–24.7)	6.3 (0.4–24.7)	6.3 (0.4–24.7)	6.3 (0.4–24.7)	0 (0)	0 (0)

Abbreviations: CI, confidence interval; CRNMB, clinically relevant nonmajor bleeding.
[a]Cancer in penis, prostate, and testicles.
[b]Cancer in kidneys, bladder, and urethra.

 © 2024. The Author(s).

Supplementary Table S4 Major bleeding according to bleeding site, anticoagulant agent, and cancer site

	Types of major bleeding									
Cancer site according to anticoagulants	Abdominal	Epistaxis	Gastrointestinal	Hematuria	Hemoptysis	Skin bleeding	Intracerebral	Vaginal	Trauma-related	Iatrogenic/postoperative
**LMWH**										
Lower gastrointestinal tract, *n*	1	0	7	1	0	0	2	0	1	0
Male genitalia[a], *n*	0	0	1	1	0	0	1	0	0	0
Respiratory or mediastinal, *n*	0	0	0	0	1	0	0	0	1	0
Hematological, *n*	0	0	0	0	0	1	0	0	0	0
Breast, *n*	0	0	1	0	1	0	0	1	0	1
Hepatobiliary and pancreatic, *n*	2	1	1	0	0	0	0	0	0	1
Gynecological, *n*	0	0	2	0	0	0	0	1	0	0
Urinary tract[b], *n*	1	1	0	0	0	0	1	0	0	1
Skin, bone, and other connective tissues, *n*	1	0	0	1	0	0	1	0	2	0
Upper gastrointestinal tract, *n*	0	0	1	0	0	0	0	0	0	0
Secondary or unspecified, *n*	1	0	0	0	0	0	0	0	0	0
Multiple primary sites, *n*	0	0	0	0	0	0	1	0	0	1
**DOAC**										
Gynecological, *n*	0	0	0	1	0	0	0	0	0	0
Skin, bone, and other connective tissues, *n*	0	0	1	0	0	0	0	0	0	0
Respiratory or mediastinal, *n*	0	0	0	0	0	0	1	0	0	0
**VKA**										
Lower gastrointestinal tract, *n*	0	0	1	0	0	0	0	0	0	0
**Other types of treatment**										
Hematological, *n*	0	0	1	0	0	0	0	0	0	0

Abbreviations: DOAC, direct oral anticoagulant; LMWH, low molecular weight heparin; VKA, vitamin K antagonist.
[a]Cancer in penis, prostate, and testicles.
[b]Cancer in kidneys, bladder, and urethra.

 © 2024. The Author(s).

Supplementary Table S5 Clinically relevant nonmajor bleeding according to bleeding site, anticoagulant agent, and cancer site

	Types of clinically relevant nonmajor bleeding								
**Cancer site according to anticoagulants**	**Epistaxis**	**Gastrointestinal**	**Hematuria**	**Hemoptysis**	**Skin bleeding**	**Vaginal**	**Trauma-related**	**Iatrogenic/ postoperative**	**Other types**
**LMWH**									
Lower gastrointestinal tract, *n*	0	3	4	1	0	0	0	0	0
Male genitalia[a], *n*	0	0	2	0	0	0	1	1	0
Respiratory or mediastinal, *n*	0	1	0	1	1	0	0	3	0
Hematological, *n*	0	1	0	0	1	0	0	0	0
Breast, *n*	0	1	1	0	1	0	0	0	2
Hepatobiliary and pancreatic, *n*	0	1	1	0	0	0	0	2	0
Gynecological, *n*	1	1	1	0	0	3	0	0	0
Urinary tract[b], *n*	0	0	4	0	1	0	0	0	0
Skin, bone, and other connective tissues, *n*	0	0	0	0	0	0	0	1	0
Upper gastrointestinal tract, *n*	0	1	2	0	0	0	0	0	0
**DOAC**									
Lower gastrointestinal tract, *n*	0	2	0	0	0	0	0	0	0
Male genitalia[a], *n*	1	0	1	0	0	0	0	0	0
Breast, *n*	0	0	1	0	0	0	0	0	0
Skin, bone, and other connective tissues, *n*	1	0	0	0	0	0	0	0	0
Urinary tract[b], *n*	0	0	1	0	0	0	0	0	1
Multiple primary sites, *n*	0	0	1	0	0	0	0	0	0
Respiratory or mediastinal, *n*	0	0	0	1	0	0	0	0	0
**VKA**									
Lower gastrointestinal tract, *n*	0	0	0	0	0	0	1	0	0
Male genitalia[a], *n*	0	1	0	0	0	0	0	0	0
Urinary tract[b], *n*	0	0	0	1	0	0	0	0	0
**Other types of treatment**									
Male genitalia[a], *n*	0	0	1	0	0	0	0	0	0
Respiratory or mediastinal, *n*	0	0	1	0	0	0	0	0	0

Abbreviations: DOAC, direct oral anticoagulant; LMWH, low molecular weight heparin; VKA, vitamin K antagonist.
[a]Cancer in penis, prostate, and testicles.
[b]Cancer in kidneys, bladder, and urethra.

 © 2024. The Author(s).
